# Graphene oxide/metal nanocrystal multilaminates as the atomic limit for safe and selective hydrogen storage

**DOI:** 10.1038/ncomms10804

**Published:** 2016-02-23

**Authors:** Eun Seon Cho, Anne M. Ruminski, Shaul Aloni, Yi-Sheng Liu, Jinghua Guo, Jeffrey J. Urban

**Affiliations:** 1 The Molecular Foundry, Materials Sciences Division, Lawrence Berkeley National Laboratory, 1 Cyclotron Road, Berkeley, California 94720, USA; 2 The Advanced Light Source, Lawrence Berkeley National Laboratory, Berkeley, California 94720, USA

## Abstract

Interest in hydrogen fuel is growing for automotive applications; however, safe, dense, solid-state hydrogen storage remains a formidable scientific challenge. Metal hydrides offer ample storage capacity and do not require cryogens or exceedingly high pressures for operation. However, hydrides have largely been abandoned because of oxidative instability and sluggish kinetics. We report a new, environmentally stable hydrogen storage material constructed of Mg nanocrystals encapsulated by atomically thin and gas-selective reduced graphene oxide (rGO) sheets. This material, protected from oxygen and moisture by the rGO layers, exhibits exceptionally dense hydrogen storage (6.5 wt% and 0.105 kg H_2_ per litre in the total composite). As rGO is atomically thin, this approach minimizes inactive mass in the composite, while also providing a kinetic enhancement to hydrogen sorption performance. These multilaminates of rGO-Mg are able to deliver exceptionally dense hydrogen storage and provide a material platform for harnessing the attributes of sensitive nanomaterials in demanding environments.

## Abstract (web summary)

Hydrogen fuel cell electric vehicles are poised to transform the automotive industry, but the lack of safe, high density solid state hydrogen storage solutions is stifling progress. Here, the authors develop a graphene oxide/magnesium nanocomposite which appears to overcome many of the existing challenges.

## Graphical abstract

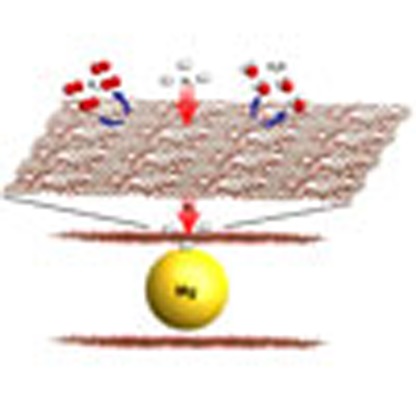

The established environmental impacts resulting from fossil fuels have stimulated urgent efforts to decarbonize our fuel sources. Hydrogen is the ultimate carbon-free energy carrier—it possesses the highest energy density among chemical fuels and water is the sole combustion product. Although major car manufacturers have made commitments to hydrogen as a ‘fuel of the future'^1^, hydrogen storage for FCEVs (fuel cell electric vehicles) currently relies on compressed gas tanks^2^. These are unable to meet long-term storage targets and severely compromise on-board occupancy. Metal hydrides for solid-state hydrogen storage are one of the few materials capable of providing sufficient storage density required to meet these long-term targets. However, simultaneously meeting gravimetric, volumetric, thermodynamic and kinetic requirements has proven challenging, owing to the strong binding enthalpies for the metal hydride bonds, long diffusion path lengths and oxidative instability of zero-valent metals. Although nanostructuring has been shown to optimize binding enthalpies^3^, synthesis and oxidative stabilization of metallic nanocrystals remains a challenge^4^. Protection strategies against oxidization and sintering of nanocrystals often involve embedding these crystals in dense matrices, which add considerable ‘dead' mass to the composite, in turn decreasing gravimetric and volumetric density. Thus, although metal hydrides show the most promise for non-cryogenic applications, it remains true that no single material has met all of these essential criteria^5^,^6^.

Here we demonstrate mixed dimensional reduced graphene oxide (GO)/Mg nanocrystal hybrids as a novel high-performance materials platform for solid-state hydrogen storage. After the first report of the preparation of individual graphene sheets in 2004 (refs 7, 8), its unique optoelectronic properties attracted great attention. GO, formerly considered just a precursor for the synthesis of graphene, has begun to find independent applications in water purification and gas separations due to its hydrophilicity, chemical structure and atomistic pore size diameters^9^,^10^. For example, GO membranes have recently been explored as materials for crucial gas separation challenges. Interestingly, these studies have shown extreme permeability for H_2_ relative to other atmospheric gases such as O_2_ and N_2_, thus providing a potential avenue for use as an atomically thin, selective barrier layer for sensitive hydrogen storage materials. Furthermore, related studies have shown that reduction of GO to form reduced GO (rGO) further results in a dramatic decrease in water permeance, while maintaining desirable gas permeability characteristics^11^. In this study, we have prepared mixed dimensional laminates of two-dimensional (2D) rGO filled with Mg nanocrystals for hydrogen storage applications (Fig. 1a). In this composite, rGO serves as the atomic limit for barrier layer materials in functional composites, providing maximum environmental protection for the least possible amount of inactive mass—theoretically, for single sheet rGO protection, up to 98 wt% of the composite mass can be active Mg—thus, this rGO composite approach yields the greatest performance in selective hydrogen permeability and kinetic enhancement in hydrogen storage. As illustrated in Fig. 1a, rGO sheets function as a protective layer against Mg nanocrystal oxidation by preventing the permeation of O_2_ and H_2_O, while still allowing hydrogen to easily penetrate, diffuse along the layers and be released. Moreover, beyond the crucial gas barrier behaviour, we demonstrate that the rGO layers add functionality to the laminates by reducing the activation energies associated with hydrogen absorption and desorption, key kinetically limiting steps for traditional metal hydride systems. Several studies have shown that carbon-based materials such as carbon fibres, nanotubes and graphite exhibit a beneficial catalytic effect on the kinetics and cyclability of hydrogen absorption and desorption of metal hydrides^12^,^13^,^14^. Although there are other reports using graphitic materials in composites for Li-ion battery applications, to our knowledge there have been no reports that take advantage of both the unique catalytic properties and high variability in gas permeability of rGO to synergistically yield new functionality. For the nanolaminate system presented, rGO layers are ideal encapsulating materials: they provide atomically thin structure to minimize added mass, catalytically enhanced rate-limiting hydrogen absorption/desorption events and protective barriers to prevent degradation of Mg nanocrystals.

## Results

### Synthesis and characterization of rGO-Mg multilaminates

**Figure 1:**
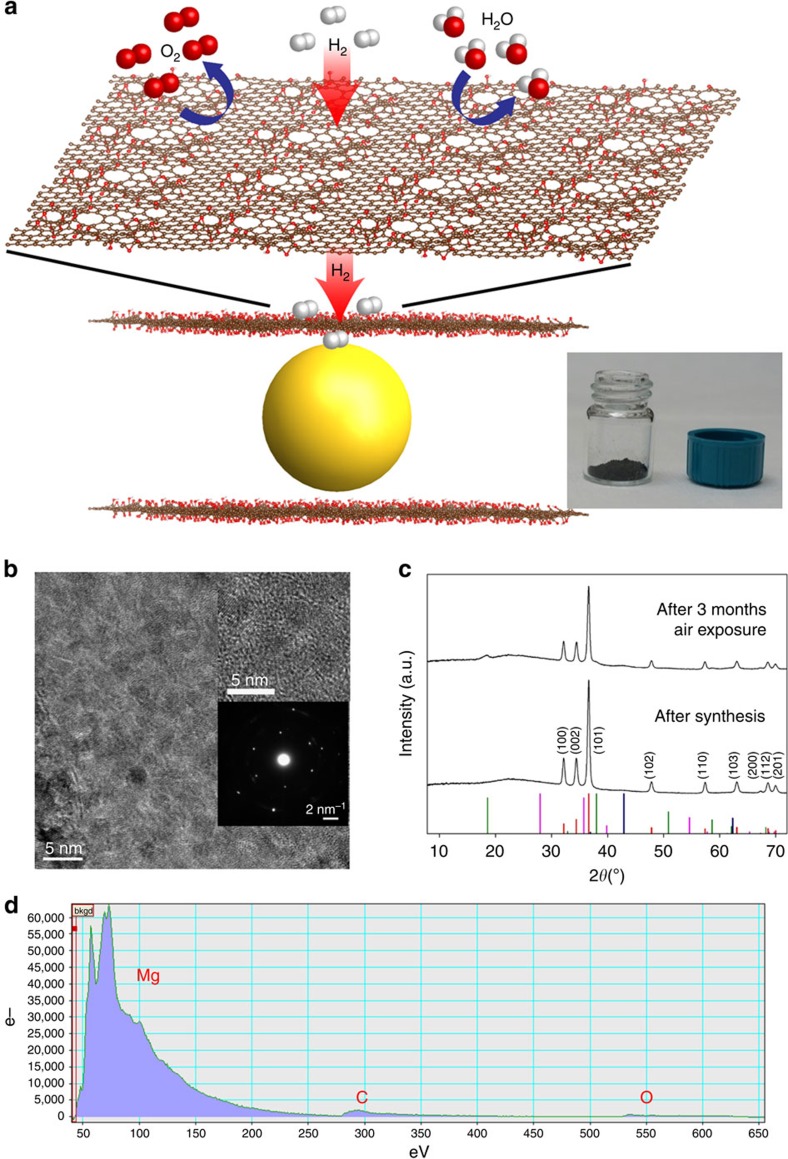
Mixed dimensional rGO-Mg laminates for stable and energetically-dense hydrogen storage. (**a**) Illustrations depicting the structure of the rGO-Mg nanolaminates where rGO layers prevent O_2_ and H_2_O from penetrating, while allowing the diffusion of H_2_ (inset: photograph of rGO-Mg in air). (**b**) TEM images of rGO-Mg showing the high density of Mg nanocrystals with no visible aggregates. The upper inset is a high-resolution image and the lower inset is diffraction pattern where the hexagonal dots are matched to Mg (100), corresponding to a *d*-spacing of 2.778 Å (JCPDS 04-0770). (More TEM images and analysis are provided in Supplementary Figs 1 and 2.) (**c**) XRD spectra of rGO-Mg after synthesis and after 3 months in air with indices of peaks (JCPDS 04-0770; the bottom bars represent an XRD pattern of hexagonal Mg (hcp) (red), tetragonal MgH_2_ (pink), hexagonal Mg(OH)_2_ (green) and cubic MgO (blue)). (**d**) EELS spectrum of a representative rGO-Mg composite flake suspended over a hole in the TEM support grid. The spectrum shows a dominant Mg *L*-edge peak and a carbon *K*-edge peak, indicating a large quantity of Mg crystals within the rGO support.

The majority of reported composites consisting of metals and carbon materials are prepared via ball milling or solidification with either polymers or carbon frameworks. However, ball-milled materials are notoriously polydisperse, which introduces corresponding inhomogeneity in properties. Moreover, such energy-intensive processes can intrinsically introduce unwanted morphological disruptions and chemical inhomogeneities, all of which detract from performance. By contrast, we have developed a direct, one-pot co-reduction, thus simultaneously forming both pristine, monodisperse nanocrystals and the desired rGO without energy-intensive processing or ligand chemistries. Observing that current approaches for reduction of GO and reduction of metal precursors to form Mg nanocrystals both rely on similar methodologies^4^,^15^,^16^,^17^, we synthesized rGO-Mg nanocomposites via a facile solution-based co-reduction method. In this process, the Mg^2+^ precursor is stabilized by GO and both of them are reduced by lithium naphthalenide. The obtained rGO-Mg was characterized via transmission electron microscopy (TEM) and X-ray diffraction (XRD), as shown in Fig. 1b,c. Mg nanocrystals were 3.26 nm diameter (±0.87 nm) based on TEM images (Supplementary Figs 1 and 2), presenting fine monodisperse nanocrystals, compared with other metal hydrides prepared by conventional method such as ball milling. Although Scherrer analysis of the XRD peak width indicates larger crystallite size of ∼15 nm (Supplementary Table 1), hundreds of TEM images over dozens of samples consistently show 3.26-nm-sized Mg nanocrystals. The observation of individual nanocrystals in TEM was challenging due to the low electron density of Mg, although in our materials Mg nanocrystals of diameter 3–4 nm are present and confirmed by multiple characterization methods. However, it is probable that there exist some clusters and networks of Mg particles in the sample as the XRD results imply the possible existence of a minor fraction comprising agglomerations of nanocrystals; as we discuss later, the H_2_ storage properties are most consistent with ∼few nanometre-sized crystallites. The area of the peaks in electron energy loss spectroscopy (EELS) measurements (Mg >>>C>O) indicated a high density of Mg within the composite (Fig. 1d). In addition, despite containing a highly dense packing of Mg nanocrystals, the nanolaminates were observed to be remarkably environmentally stable. To investigate the limits of stability, rGO-Mg samples were exposed to air and characterized over time by XRD and TEM (Fig. 1c and Supplementary Fig. 1); remarkably, even after 3 months of air exposure, the nanocrystals remained almost entirely zero-valent crystalline Mg, while showing invasion of only a low-intensity Mg(OH)_2_ peak (Supplementary Fig. 3 and an additional corrosion test result in Supplementary Fig. 4 and Supplementary Discussion). Moreover, to show the reliability offered by this approach, we completely exposed the sample to air and then demonstrated hydrogen cycling. This is not possible with any other hydride technology with comparable storage density. Although none of the techniques used for characterization show evidence for extensive formation of oxides of Mg (MgO or Mg(OH)_2_), it is probable that a thin, self-terminating layer of oxide or sub-oxide may form on the nanocrystals; however, this oxide does not measurably have an impact on performance (Fig. 1d and Supplementary Fig. 5).

### Hydrogen capacity and kinetic analysis

**Figure 2:**
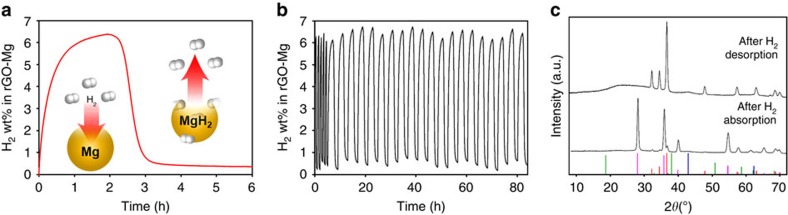
Hydrogen absorption/desorption characterization of rGO-Mg multilaminates. (**a**) Hydrogen absorption/desorption (at 200 °C and 15 bar H_2_/300 °C and 0 bar) for the prepared rGO-Mg multilaminates. (**b**) Hydrogen absorption/desorption cycling of rGO-Mg multilaminates that were first exposed to air overnight. The first 5 cycles were performed at 250 °C and 15 bar H_2_/350 °C and 0 bar, and the additional 20 cycles at 200 °C and 15 bar H_2_/300 °C and 0 bar. (**c**) XRD spectra of rGO-Mg after absorption/desorption (the bottom bars represent the XRD patterns of Mg (red), MgH_2_ (pink), Mg(OH)_2_ (green) and MgO (blue)).

Hydrogen absorption and desorption characteristics of the rGO-Mg composite were tested using a Sieverts PCT-Pro instrument at 15 bar H_2_ and 0 bar, respectively, as shown in Fig. 2a. Hydrogen uptake was immediate and the formation of MgH_2_ was confirmed by XRD (Fig. 2c) and electron diffraction (Supplementary Fig. 1). The hydrogen absorption capacity of the composite was 6.5 wt% and 0.105 kg H_2_ per litre in the total composite, which is the highest capacity reported (calculated on a full composite mass basis) using metal hydrides under the comparable conditions. This corresponds to 7.56 wt% H_2_ in Mg nanocrystals, which is 99.5% of the theoretical value (7.6 wt%)^5^. Given the atomically thin nature of the encapsulation, these nanocomposites achieve denser packing of metal nanocrystals than is possible by any competing approach, leading to optimized storage density. Furthermore, hydrogen also readily desorbed up to 6.12 wt% in the composite, thus demonstrating excellent reversibility. To verify that the hydrogen absorption was not caused by the presence of GO in the composite, control studies using only GO were conducted and exhibited minimal (<0.2 wt% in GO) absorption at 200 °C and 250 °C (Supplementary Fig. 6). This is a negligible contribution, given that the amount of GO in the multilaminates is <2 wt% overall. To analyse the kinetics, the activation energy (*E*_a_) for hydrogen absorption/desorption was determined from measurements at three different temperatures, fitting the result with the Johnson–Mehl–Avrami model (details are described in Supplementary Discussion, Supplementary Figs 7 and 8, and Supplementary Table 2)^18^. The *E*_a_ values were 60.8 and 92.9 kJ mol^−1^ for absorption and desorption, respectively, consistent with one-dimensional nucleation and growth as shown previously^4^,^19^. Remarkably, these kinetics are comparable to transition metal-catalysed bulk metal-hydride systems, and the overall capacity and kinetics greatly surpass the best environmentally robust samples made up to date^4^,^17^. We ascribe the performance of our composite to the unique features of this multilaminate: the nanoscale size of the Mg crystals is comparable to molecular diffusion lengths, which enables near-complete conversion to the metal hydride (99.5% of the theoretical value) and the interaction of the Mg nanocrystals with the rGO layers protects against invasion of oxygen, while enabling rapid surface diffusion of hydrogen, which enhances kinetics^20^. Indeed, rGO-Mg hydrogen absorption/desorption is faster than Mg-polymer composites containing nanocrystals of similar size (Supplementary Fig. 9 and Supplementary Discussion). Consistent with previous studies, the diffusion of hydrogen atoms was facilitated by the interaction between Mg and carbon layers, enhancing both the hydrogen capacity and kinetics of Mg (additional data in Supplementary Fig. 10 and Supplementary Discussion)^21^,^22^. Cycle tests were performed at 250 °C/350 °C for 5 cycles and at 200 °C/300 °C for additional 20 cycles (Fig. 2b). Importantly, each cycle test was conducted successively to better mimic real-world fueling/re-fueling conditions without manual evacuation in between each cycle (additional experimental results in Supplementary Fig. 11). The capacity and kinetics were well preserved during further cycles, although a slight decrease in the capacity was observed. Noticeably, the Mg nanocrystal size and size distributions were well preserved after several absorption/desorption cycles without sintering or grain growth (Supplementary Fig. 1). Although bulk metal hydrides are susceptible to mechanical fracture and cracking due to the large volume expansion on hydriding (*ca*. 33% from Mg to MgH_2_), the high Young's modulus of rGO enables it to robustly encase the Mg nanocrystals during expansion/contraction events without fracture and prevent macroscale sintering.

### Structural analysis of rGO-Mg composite

**Figure 3:**
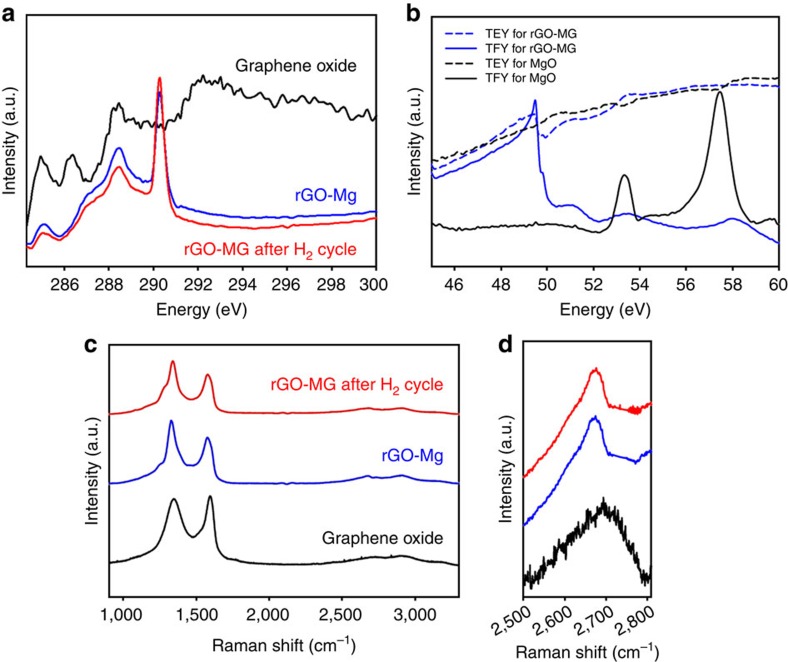
XANES and Raman spectral analysis of GO and rGO-Mg multilaminates before and after hydrogen cycling. XANES spectra of (**a**) GO, rGO-Mg after synthesis and after cycling at carbon *K*-edge, and (**b**) rGO-Mg after synthesis and MgO at Mg *L*-edge. Raman spectra of (**c**) GO, rGO-Mg after synthesis and after H_2_ cycling, and (**d**) the 2D peak region.

**Figure 4:**
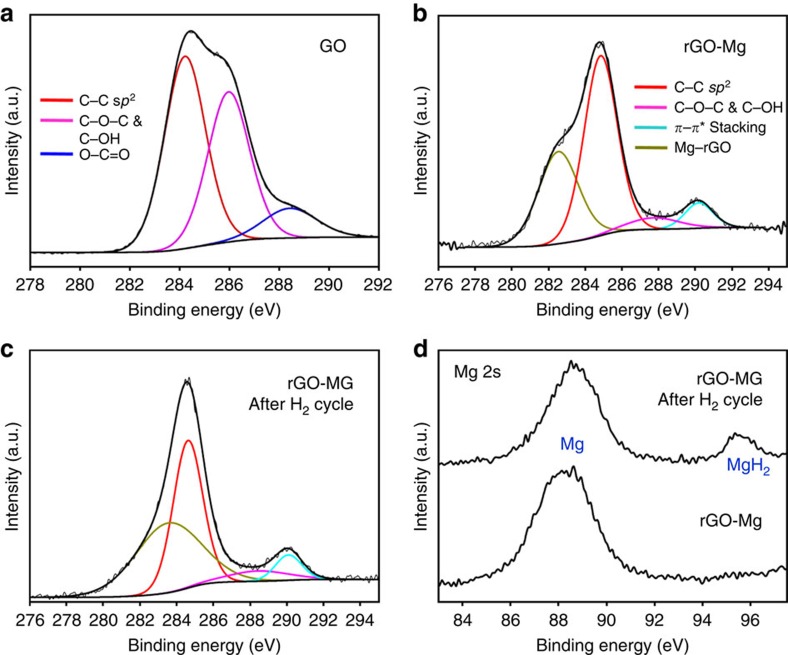
X-ray photoelectron spectra (XPS) of rGO-Mg after synthesis and after hydrogen cycling. XPS spectra (C, 1 s) of (**a**) GO, (**b**) rGO-Mg after synthesis and (**c**) rGO-Mg after H_2_ cycling. (**d**) XPS pattern (Mg, 2 s) for rGO-Mg after synthesis and after H_2_ cycling.

X-ray absorption near-edge structure (XANES) measurements were performed to probe the interactions between rGO and Mg nanocrystals (Fig. 3a). Compared with GO, increased intensity of carbon *K*-edge at 288.4 and 290.3 eV were observed, corresponding to carbon atoms attached to oxygen or other oxygen-containing chemical species. From this, we infer that the multilaminates are uniquely stabilized by the formation of interfacial Mg–O–C bonds forged during synthesis^23^. We believe this to be the basis of the exceptional stability of these multilaminates. In addition, the zero-valent state of Mg nanocrystals was verified at Mg *L*-edge measurement as both total electron yield and total fluorescence yield scans display an explicit Mg metal peak (49.8 eV) without distinct MgO peaks (Fig. 3b and additional analysis in Supplementary Fig. 5 and Supplementary Discussion), consistent with XRD and EELS spectra. The structural evolution of GO during synthesis and hydrogen cycling was studied using Raman spectroscopy (Fig. 3c,d). The intensity ratio of *D* and *G* peaks (*I*(*D*)/*I*(*G*)) increased after rGO-Mg synthesis, indicating that the average domain size of *sp*^2^ hybridized regions was decreased as GO was reduced^24^. The 2D peak, whose position and shape depends on the number of graphene layers, shifted to lower frequency (2,701 cm^−1^ to 2,685 cm^−1^) and its full width at half maximum also decreased on the formation of rGO-Mg (Fig. 3d). This suggests that few, if any, isolated multilayers of rGO exist in the composite, and that most rGO layers are actively wrapping Mg nanocrystals^25^,^26^ (additional analysis in Supplementary Fig. 12 and Supplementary Discussion). No change was observed in the Raman spectra of freshly synthesized rGO-Mg in comparison with samples studied after hydrogen cycling. Importantly, *I*(*D*)/*I*(*G*) ratios remained consistent as well (1.370 after synthesis and 1.337 after cycling), indicating that the defect density, a key attribute of rGO responsible for selective hydrogen transport, was well maintained even after several hydrogen absorption and desorption cycles^25^. In addition, the chemical environment of GO and rGO-Mg were investigated via X-ray photoelectron spectroscopy (Fig. 4). Peaks associated with oxygen-containing functional groups in the GO diminished after the formation of rGO-Mg, confirming reduction of GO^27^. The rGO-Mg composite contained an additional peak at 282.5 eV, which is attributed to the interaction between carbon species and metal particles^28^,^29^, corresponding to the interaction of rGO and Mg nanocrystals. Furthermore, a prominent *π*–π* stacking peak was observed at 290.1 eV, resulting from Mg nanocrystal wrapping, which was also observed by TEM (Supplementary Fig. 1). In the Mg 2s spectrum, one additional peak appears in the higher energy region after hydrogen cycling, implying a new chemical state, consistent with MgH_2_ (ref. 30). The Mg and MgH_2_ contents were 91.45% and 8.55%, respectively, after completing cycle test based on Mg 2s spectrum, explaining a slight amount of the remaining hydride phase in the end of cycling.

## Discussion

We have developed a facile method of preparing the densest possible loading of reactive nanocrystals safely into a composite material, a crucial step forward for enhancing the energy density of nanomaterials. As a result, our rGO-Mg multilaminates offer exceptional environmental stability and unsurpassed hydrogen storage capability, exceeding that offered by any other non-cryogenic reversible material. We believe that these results suggest the possibility of practical solid-state hydrogen storage and use in the near future. Furthermore, this work shows that atomically thin 2D materials can be used to simultaneously protect nanocrystals from ambient conditions, while also imparting new functionality. Such stable mixed dimensional laminates of zero-valent nanocrystalline metals can be extended to a variety of additional applications, including batteries, catalysis, encapsulants and energetic materials.

## Methods

### Synthesis of rGO-Mg nanocomposite

The composites of rGO-Mg were synthesized in an argon glove box. GO was ball milled for 10 min before use, to break it down to GO platelets so that it can effectively make a complex with bis(cyclopentadienyl) magnesium (Cp_2_Mg). To prepare the lithium naphthalenide solution, naphthalene (2.40 g, 0.0187, mol) was dissolved in 120 ml of tetrahydrofuran (THF), followed by the immediate addition of Li metal (0.36 g, 0.0253, mol), leading to a dark green solution. GO (6.25 mg) was dispersed in 12.5 ml of THF under Ar, sealed in a container and sonicated for 1.5 h. A Cp_2_Mg solution (2.31 g, 0.015 mol, in 22.5 ml of THF) was added to GO solution afterwards, stirring for 30 min. The resulting GO/Cp_2_Mg solution was added to the lithium naphthalenide solution and magnetically stirred for 2 h. The product was centrifuged (10,000 r.p.m., 20 min) and washed with THF (10,000 r.p.m., 20 min) twice, followed by vacuum drying overnight.

### Characterization and instrumentation

High-resolution TEM was performed using JEOL 2100-F Field-Emission Analytical Transmission Electron operated at 120 kV and equipped with Oxford INCA energy-dispersive electron X-ray spectrometer and Tridiem Gatan imaging Filter and spectrometer. The powder samples were dispersed on lacey carbon grids from THF solutions. Elemental analysis of the EELS and energy-dispersive spectra was performed using Digital Micrograph software (Gatan Inc.). XRD patterns were acquired with a Bruker AXS D8 Discover GADDS X-Ray Diffractometer, using Cu Kα radiation (*λ*=0.154 nm). Hydrogen absorption/desorption measurement was performed, using a HyEnergy PCT Pro-2000 at 15/0 bar of H_2_ at different temperatures. XANES spectroscopy was performed on Beamline 8.0.1.3 and 4.0.3 at the Advanced Light Source. The energy resolution at carbon *K*-edge and Mg *L*-edge was set to 0.1 eV and the experimental chamber had a base pressure of at most 1 × 10^−8^ torr. An highly ordered pyrolytic graphite (HOPG) reference sample was measured before and after all XANES experiments for energy calibration. The XANES spectra were recorded using total electron yield and total fluorescence yield detection modes. The Raman spectra of GO and rGO-Mg samples were collected using Horiba Jobin Yvon LabRAM ARAMIS automated scanning confocal Raman microscope with a 532-nm excitation source and X-ray photoelectron spectra were obtained via PHI 5400 X-ray Photoelectron Spectroscopy System with Al Kα. The Mg content in the composite was determined by inductively coupled plasma-optical emission spectroscopy at the Advanced Light Source Life Sciences Division and Environmental.

## Additional information

**How to cite this article**: Cho, E. S. *et al*. Graphene oxide/metal nanocrystal multilaminates as the atomic limit for safe and selective hydrogen storage. *Nat. Commun.* 7:10804 doi: 10.1038/ncomms10804 (2016).

## Supplementary Material

Supplementary Information Supplementary Figures 1-12, Supplementary Tables 1-2, Supplementary Note 1, Supplementary Discussion and Supplementary References
