# Erratum: Graphene oxide/metal nanocrystal multilaminates as the atomic limit for safe and selective hydrogen storage

**DOI:** 10.1038/ncomms11145

**Published:** 2016-03-18

**Authors:** Eun Seon Cho, Anne M. Ruminski, Shaul Aloni, Yi-Sheng Liu, Jinghua Guo, Jeffrey J. Urban

Nature Communications 7:10804 Article number 1080410.1038/ncomms10804 (2016); Published 02232016; Updated 03182016

The financial support for this Article was not fully acknowledged. The Acknowledgements should have included the following:

The authors gratefully acknowledge research support from the U.S. Department of Energy, Office of Energy Efficiency and Renewable Energy, Fuel Cell Technologies Office, under Contract No. DE-AC02-05CH11231.

