# Supplementary material for: Graphene oxide/metal nanocrystal multilaminates as the atomic limit for safe and selective hydrogen storage
Source: Nat Commun. 2016 Feb 23;7:10804. doi: 10.1038/ncomms10804 (PMC4766423; doi:10.1038/ncomms10804)
Supplement: Supplementary Information — Supplementary Figures 1-12, Supplementary Tables 1-2, Supplementary Note 1, Supplementary Discussion and Supplementary References [file ncomms10804-s1.pdf]

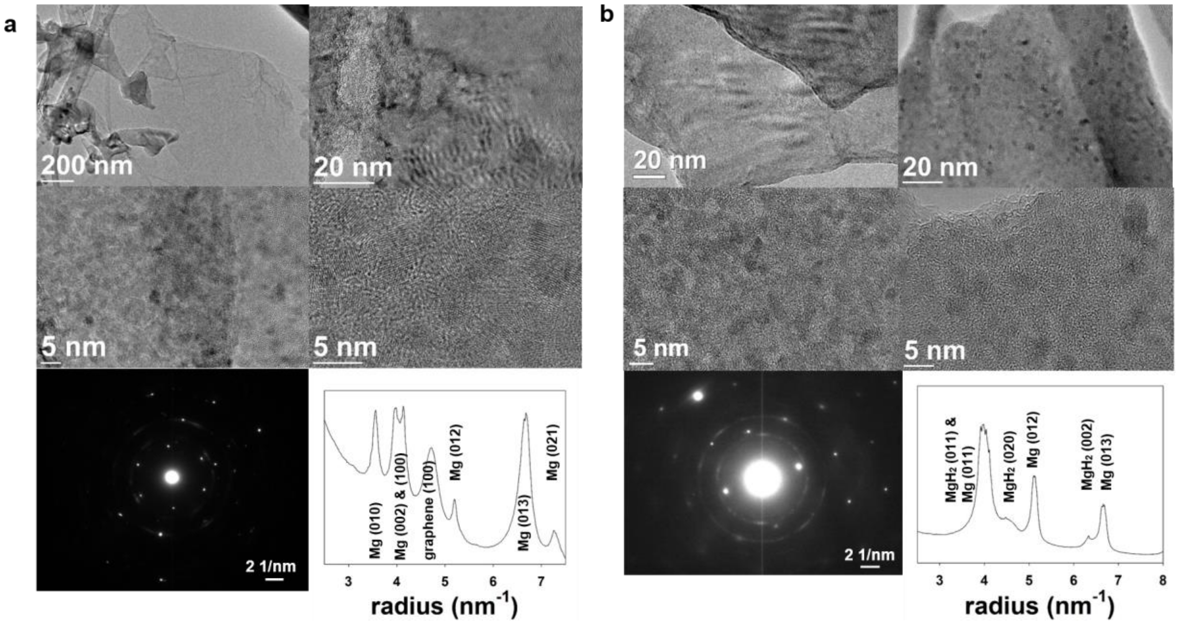

Supplementary Figure 1. TEM images of rGO-Mg **a.** after synthesis and **b.** after hydrogen cycling. The diffraction patterns were analyzed via Image J Radial Profile Angle software, which produces a plot of normalized integrated radial intensities; the corresponding plot is shown here in the lower right hand panel.

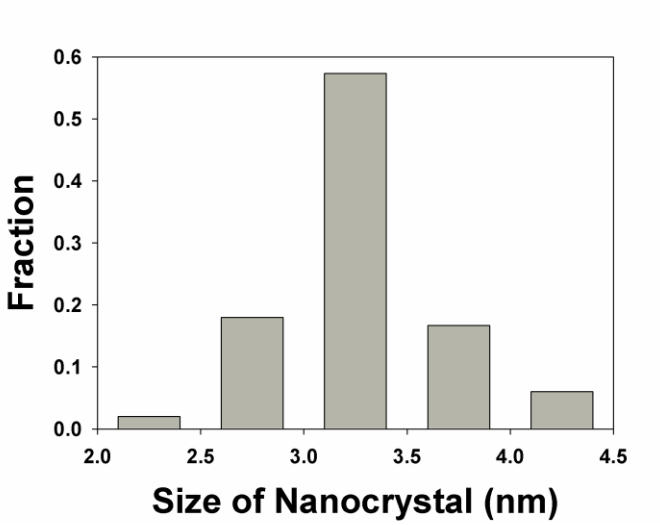

Supplementary Figure 2. Histogram of Mg nanocrystal size distribution (3.26 nm diameter (±0.87 nm)) as determined by TEM of hundreds of crystallites.

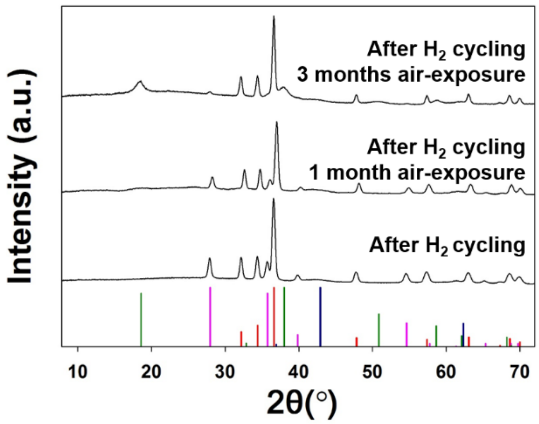

Supplementary Figure 3. XRD spectra of the composite after cycling (5 cycles) with partial desorption and subsequent air exposure (The bottom bars represent a XRD pattern of Mg (red), MgH_2 (pink), $Mg(OH)_2$ (green), MgO (blue).).

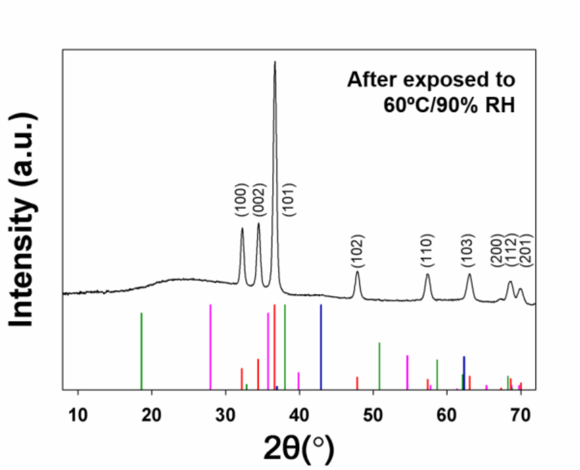

Supplementary Figure 4. XRD spectra of rGO-Mg after exposed to 60 ºC/90% RH in the environmental chamber with indices of peaks (The bottom bars represent a XRD pattern of Mg (red), MgH_2 (pink), $Mg(OH)_2$ (green), MgO (blue).) (see the Supplementary Discussion).

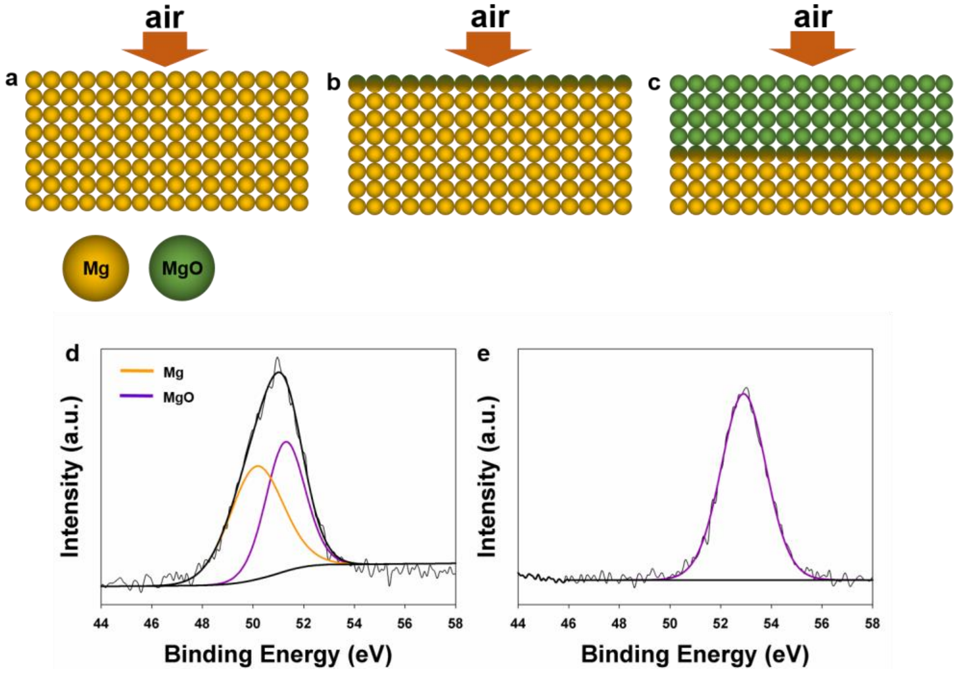

Supplementary Figure 5. Illustrations representing three possible oxidation state of Mg nanocrystals in the composite upon exposure to air: **a.** pure Mg metal without any oxidation, **b.** a very thin MgO layer formation underneath the interface with air, mostly maintaining the Mg metal state in bulk (approximately 0.5 wt% for a 3.26 nm spherical nanocrystal), **c.** the existence of a large amount of MgO as a result of extensive oxidation, and XPS spectra (Mg 2p) for **d.** rGO-Mg and **e.** MgO powder (see the Supplementary Discussion).

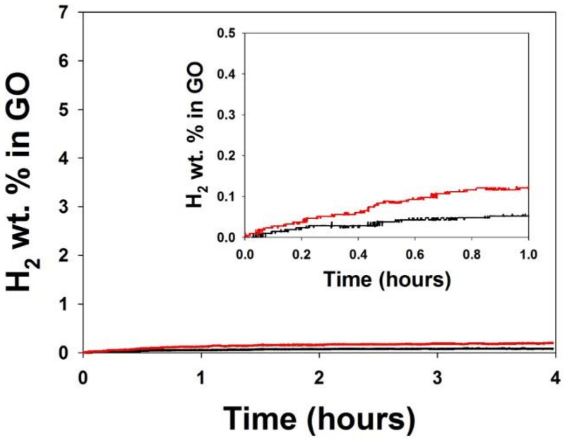

Supplementary Figure 6. Hydrogen absorption of GO: Black and red lines represent hydrogen absorption at 200 ºC and 250 ºC, respectively, for 4 hours at 15 bar $H_{2.}$ (The inset shows a magnified version for the first hour of absorption.)

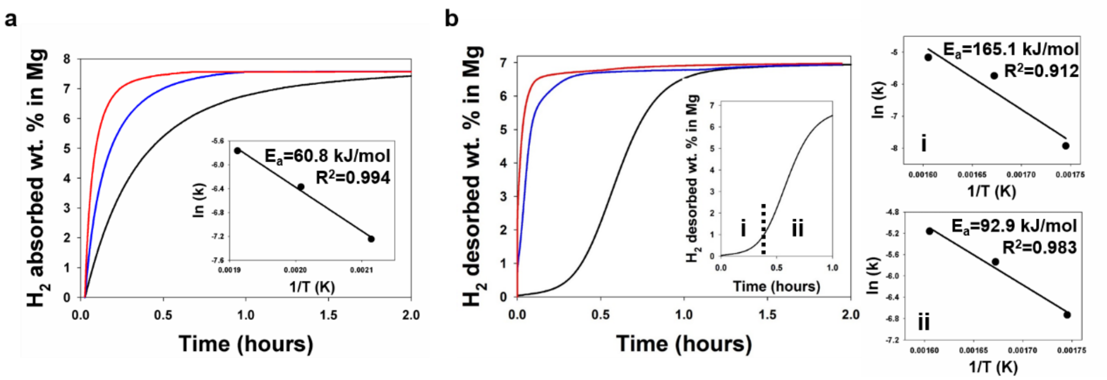

Supplementary Figure 7. a. Hydrogen absorption at three different temperatures (black: 200 ºC, blue: 225 ºC, red: 250 ºC) at 15 bar H_2, **b.** Hydrogen desorption at three different temperatures (black: 300 ºC, blue: 325 ºC, red: 350 ºC) at 0 bar. The inset shows two different desorption regions at 300 ºC (see the Supplementary Discussion).

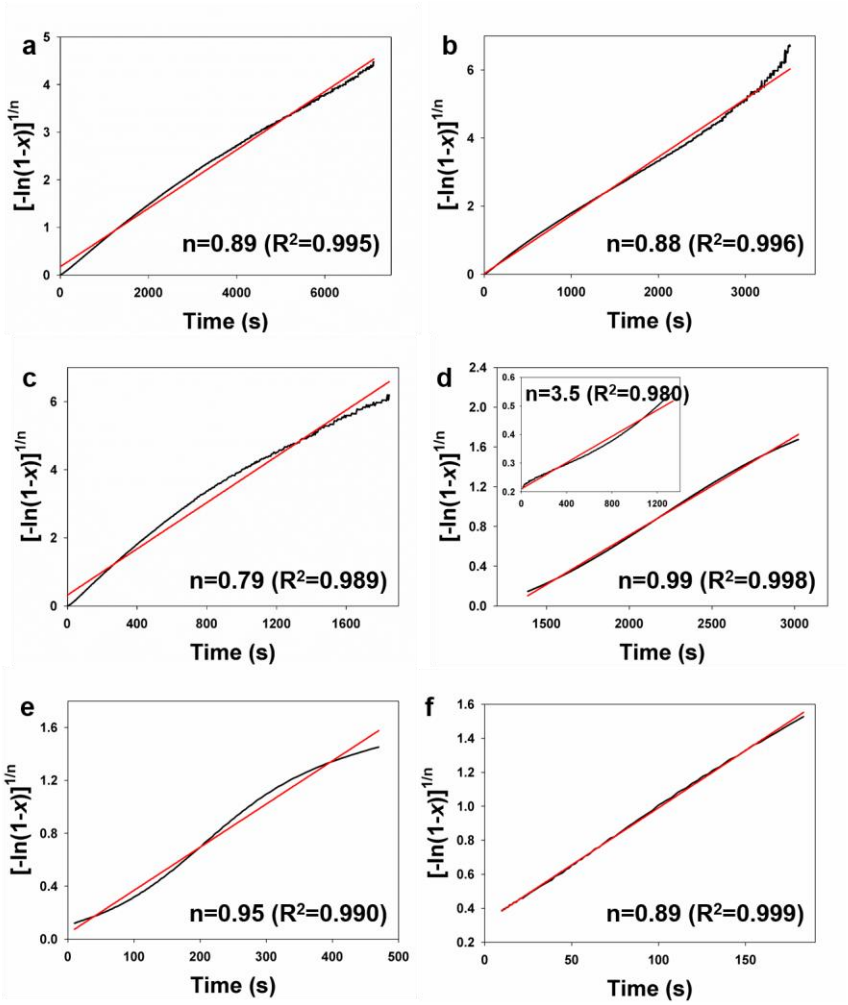

Supplementary Figure 8. JMA fitting for all absorption and desorption measurements with "best" *n* values, absorption at **a.** 200 ºC**, b.** 225 ºC**, c.** 250 ºC, and desorption at **d.** 300 ºC (the inset shows the fitting of the initial 1wt% desorption)**, e.** 325 ºC**, f.** 350 ºC (see the Supplementary Discussion).

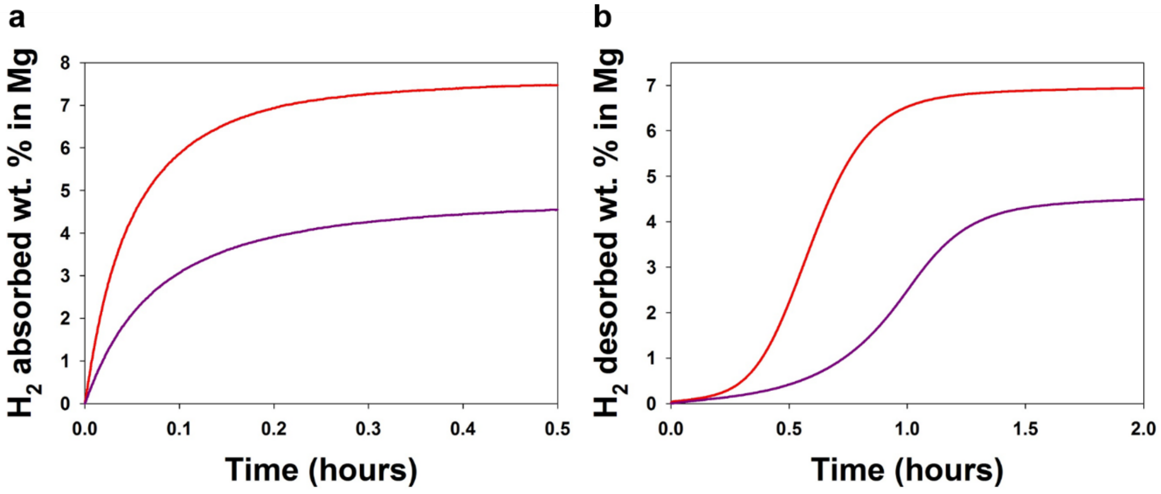

Supplementary Figure 9. a. Hydrogen absorption at 250 ºC and 15 bar H_2, **b.** Hydrogen desorption at 300 ºC and 0 bar for rGO-Mg (red) and Mg-PMMA (purple) (see the Supplementary Discussion).

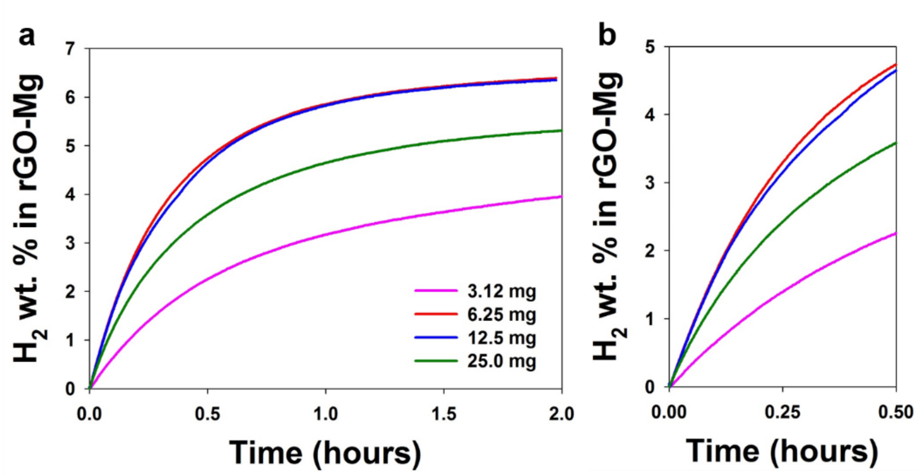

Supplementary Figure 10. a. Hydrogen absorption at 200 ºC and 15 bar H_2 with different amount of GO, as indicated (the original amount of GO discussed is 6.25 mg, as described in Methods). **b.** The first 0.5 hour of the H_2 absorption traces are magnified, better demonstrating the clear difference in kinetics (see the Supplementary Discussion).

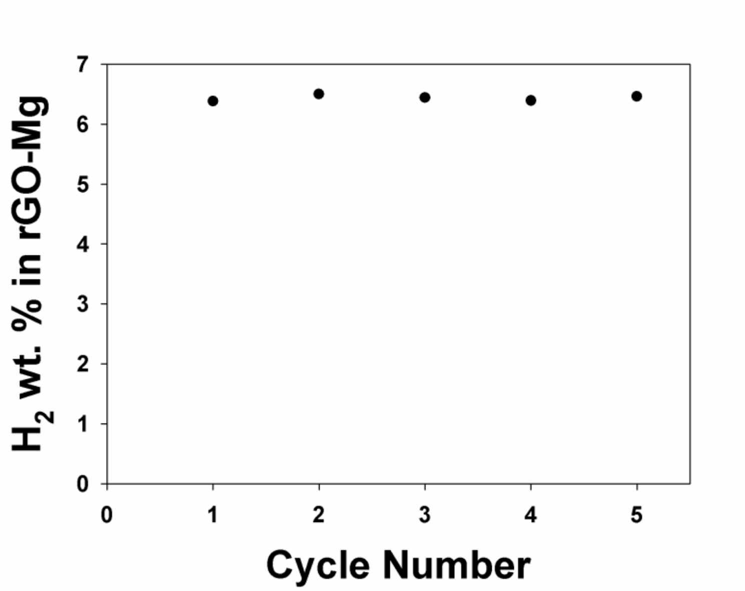

Supplementary Figure 11. Hydrogen capacity during cycles after placed under an open vacuum (see the Supplementary Note 1).

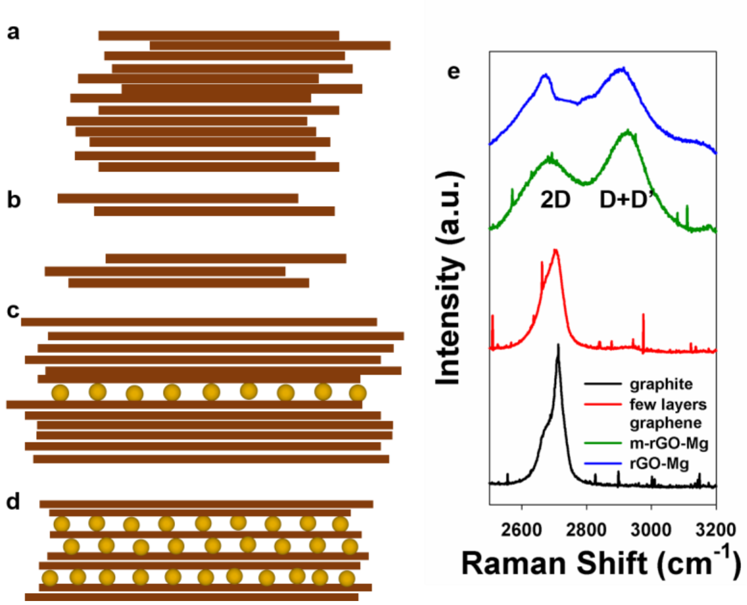

Supplementary Figure 12. Illustrations representing the structure of **a**. graphite, **b**. few layers of graphene, **c**. m-rGO-Mg, **d**. rGO-Mg, and **e**. their Raman spectra in 2D region (see the Supplementary Discussion).

Batch #	Index	2θ (degree)	β (degree)	Size (nm)
1	(100)	32.15	0.49885	16.57
	(002)	34.39	0.57451	14.47
	(101)	36.57	0.54118	15.45
2	(100)	32.03	0.61335	13.47
	(002)	34.22	0.65019	12.78
	(101)	36.43	0.67452	12.39
3	(100)	32.19	0.48416	17.07
	(002)	34.42	0.55813	14.90
	(101)	36.60	0.53017	15.78

Supplementary Table 1. Determination of the average size of Mg nanocrystals in the composite using X-ray diffraction peaks (see the Supplementary Discussion).

<table>
<tr><th rowspan="2"></th><th colspan="2">Absorption</th><th rowspan="2"></th><th colspan="2" rowspan="2">Desorption</th></tr>
<tr><th>~60% abs.</th><th>~100% abs.</th></tr>
<tr><td>200 ºC</td><td>0.98
(R^2=0.999)</td><td>0.89
(R^2=0.995)</td><td>300 ºC</td><td>3.5
(R^2=0.983)</td><td>0.99
(R^2=0.998)</td></tr>
<tr><td>225 ºC</td><td>1.00
(R^2=0.998)</td><td>0.88
(R^2=0.996)</td><td>325 ºC</td><td colspan="2">0.95 (R^2=0.990)</td></tr>
<tr><td>250 ºC</td><td>0.98
(R^2=0.998)</td><td>0.79
(R^2=0.989)</td><td>350 ºC</td><td colspan="2">0.89 (R^2=0.999)</td></tr>
</table>

Supplementary Table 2. The best *n* values in JMA fits with R^2 values

Supplementary Note

Supplementary Note 1: The desorption cycles were performed under a closed system in our experiments, leaving 0.3-0.4 wt% of the residual hydrogen; however, such residual hydrogen can be completely (>99%) dehydrided by evacuating the composite sample under an open vacuum for 30 minutes (Supplementary Fig. 11).

Supplementary Discussion

Corrosion Test for rGO-Mg: The rGO-Mg composite was placed in the environmental chamber with 60 ºC–the upper limit in the range of ambient operating temperature of FCEV by DOE–and 90% of relative humidity for 3 days to verify its safety under environmental exposure, followed by XRD measurement (Supplementary Fig. 4). Remarkably, the Mg crystalline structure was well-maintained without oxidation due to the rGO encapsulation layers.

Investigation of Oxidation State of Mg in the Composite: To best interpret our results, we postulated three possible oxidative conditions for Mg nanocrystals in the composite, as shown in Supplementary Fig. 5 a-c: a) pure Mg metal state, b) mono or few MgO layer formation underneath the interface with air, mostly maintaining the Mg metal state in bulk, c) the existence of a large amount of MgO. Both the EELS spectrum (Fig.1d) and XANES measurement at Mg L-edge (Fig. 3b) are consistent with a zero-valent Mg state, corresponding to the XRD measurement (Fig. 1c); however, a rather weak Mg peak emerges in the TEY (which is surface-sensitive) at the Mg L-edge while the TFY scan (which represents the bulk state) does not show this prominently–implies the possible existence of a very thin layer of magnesium oxidation. To elucidate the oxidation state of Mg crystals in the composite upon exposure to air, additional

XPS measurements were performed. In the Mg 2p spectrum, rGO-Mg composite had two peaks which are best deconvolved into two signals representing Mg and MgO states, respectively, while the MgO powder presented a single MgO peak. Thus, it can be inferred that a very thin– possibly less than 1 nm–MgO layer exists only on the surface of the bulk sample as the cartoon in Supplementary Fig. 5b; however, based on all experimental results, the zero-valent Mg nanocrystals are dominant and well-maintained.

Kinetic Analysis for Hydrogen Sorption of rGO-Mg: All measurements were performed with one sample, and the obtained data were fit– the full absorbed fraction and 85% of the desorbed fraction were used–with the Johnson-Mehl-Avrami equation (equation (1)),

$$[-\ln(1-\mathrm{x})]^{1/n} = kt \qquad (1)$$

where *x* is the fraction of Mg or MgH_2 hydrogenated or dehydrogenated, *k* is the reaction rate constant, *t* is time, and *n* is the reaction exponent. For the absorption measurement, the best linear behavior was acquired with *n*=0.79-0.89, though *n*=0.98-1.00 was obtained for the initial 60 % of absorption fraction (Supplementary Fig. 7,8 and Supplementary Table 2), implying that nucleation and growth along one-dimension with the formation of rod-shaped MgH_2 occurs dominantly, while some irregular shape is produced in the late stage of adsorption, as deduced from the non-integer *n* values. The activation energy of absorption was calculated to be 60.8 kJ/mol with R^2 = 0.994. For the desorption measurement, however, a different behavior was observed at 300 ºC. Unlike 325 ºC and 350 ºC, the curve shape changed upon approximately 1 wt% of H_2 desorption for 300 ºC; hence, the data at 300 ºC was separated into two regions, before and after 1 wt% desorption (labeled as region **i** and **ii**, respectively, in Supplementary Fig.

7(b) inset), for an accurate analysis. The best linear behavior was obtained with n=0.95 and 0.89 for 325 ºC and 350 ºC, respectively, while n=3.5 and n=0.99 for 300 ºC, before (**i**) and after (**ii**) 1 wt% desorption, respectively, indicating the change of mechanism. Using the two different regimes, different activation energies were obtained: 165.1 kJ/mol (R^2 = 0.912) and 92.9 kJ/mol (R^2 = 0.983) respectively. The curve fitting had a higher R^2 value when the data region with n=0.99 was used. It can be inferred that, at high temperatures, hydrogen is desorbed via rapid nucleation followed by one-dimensional growth, whereas at 300ºC, slow nucleation occurs until 1 wt% of hydrogen is desorbed, followed by one-dimensional growth.

Comparison of Hydrogen Sorption between rGO-Mg and Mg-PMMA: The hydrogen absorption/desorption properties of the nanolaminate were compared with Mg-PMMA[1] which has similarly sized Mg nanocrystals encapsulated by poly(methyl methacrylate) (PMMA) (Supplementary Fig. 9). Enhancements of both hydrogen capacity and sorption kinetics were observed for the rGO-Mg multilaminates; clearly, the presence of the rGO-layers has a beneficial effect on sorption and desorption kinetics.

The Role of rGO in Hydrogen Sorption of the Composite: The amount of GO in the composite was varied in order to examine the effect of mass fraction of rGO on sorption behavior (Supplementary Fig. 10). Interestingly, relative to the reported abundance of rGO in the manuscript, both additional and less GO in the synthesis resulted in reduced hydrogen capacity and poorer kinetics. Based upon these results, we observe that the catalytic effect of rGO on sorption was diminished when less GO was used, while a larger amount of GO could hinder hydrogen diffusion into and out of the Mg nanocrystals by increasing the diffusion path length. Consequently, there exists an optimum weight percent range of GO for optimized performance

of the nanolaminates, where rGO crucially prevents Mg nanocrystals from oxidization, while also enhancing the kinetics and maximizing hydrogen capacity.

Raman Analysis for rGO Sheet/Sheet Coupling: To study the evolution of the Raman spectra as sheet coupling is increased, Mg crystals with a large amount of rGO (m-rGO-Mg)–16 times more GO (relative to the reported synthesis) were prepared. With vastly more rGO in this m-rGO-Mg sample, rGO/rGO (e.g. sheet/sheet) interactions are prominent and Mg nanocrystals are embedded in multiple rGO layers as shown in Supplementary Fig. 12c, while they are encapsulated by a single or few layers of rGO in the structure of rGO-Mg used as shown in Supplementary Fig. 12d. The Raman spectra of both composites exhibited distinct differences with respect to that of bare graphite, as expected. As shown in Supplementary Fig. 12e, for bare graphite, the 2D peak appears at ~2710 cm^{-1}. The 2D peak region of both composites present a new band exhibiting the same characteristics as reported in few layer wrinkled graphene, and the 2D peak itself resembles few layer of graphene sheet which prepared via a scotch-tape method. As anticipated based upon theory (Supplementary Fig. 12), the m-rGO-Mg materials have more sheet-sheet interactions which soften and broaden the vibrational states, which is evident by comparing the m-rGO-Mg Raman spectra to that of rGO-Mg, which shows a narrower and more blue-shifted 2D peak. This evolution of vibrational spectra matches theoretical predictions in the graphene literature, and further confirms that peak position and breadth can be used to characterize the extent of sheet/sheet coupling in these composites.

Determination of the Average Size of Mg in the Composite Using XRD: The Scherrer Equation (equation (2)) is used to determine the size of Mg nanocrystals based on XRD result,

$$D = \frac{0.9\lambda}{\beta cos\theta} \quad (2)$$

where D is the size of crystal, λ is the X-ray wavelength (0.154 nm), β is the full width at half maximum of the diffraction peak (FWHM), and θ is Bragg angle. Three major peaks were examined from three different syntheses (Supplementary Table 1). The average size determined from XRD is 14.76 nm (±1.92 nm). It is different from TEM analysis in which case the size is 3.26 nm (±0.87 nm) calculated from several random spots, and larger crystallites corresponding to 15 nm were not observed in TEM images.

Supplementary References

1 Ruminski, A. M., Bardhan, R., Brand, A., Aloni, S. & Urban, J. J. Synergistic enhancement of hydrogen storage and air stability via Mg nanocrystal-polymer interfacial interactions. *Energ Environ Sci* **6**, 3267-3271 (2013).
